# Identification of the histone acetyltransferase gene family in the *Artemisia annua* genome

**DOI:** 10.3389/fpls.2024.1389958

**Published:** 2024-07-24

**Authors:** Yang Guo, Yan You, Furong Chen, Yong Liao

**Affiliations:** Department of Pharmacy, Second Clinical Medical College, China Three Gorges University, Yichang, Hubei, China

**Keywords:** *Artemisia annua*, gene family, histone acetyltransferases, epigenetics, medical plant

## Abstract

As the most effective therapeutic drug for malaria, artemisinin can only be extracted from *Artemisia annua* L., which is sensitive to the surrounding growing habitat. Histone acetyltransferases (HATs) contain acetyl groups, which modulate mRNA transcription and thereby regulate plant environmental adaptation. Comprehensive analyses of *HAT*s have been performed in many plants, but systematic identification of *HAT*s in medicinal plants is lacking. In the present study, we identified *11 AaHAT*s and characterized these genes into four classes according to their conserved protein structures. According to the phylogenetic analysis results, potential functions of *HAT* genes from *Arabidopsis thaliana*, *Oryza sativa*, and *A. annua* were found. According to our results, *AaHAT* has a highly conserved evolutionary history and is rich in highly variable regions; thus, *AaHAT* has become a comparatively ideal object of medical plant identification and systematic study. Moreover, motifs commonly present in histone acetyltransferases in the *A. annua* genome may be associated with functional *AaHAT*s. *AaHAT*s appear to be related to gene-specific functions. AaHATs are regulated by *cis*-elements, and these genes may affect phytohormone responsiveness, adaptability to stress, and developmental growth. We performed expression analyses to determine the potential roles of *AaHAT*s in response to three environmental stresses. Our results revealed a cluster of *AaHATs* that potentially plays a role in the response of plants to dynamic environments.

## Introduction

Post-transcriptional modifications of histones mainly occur at N-terminal tails. These modifications extend out of the so-called nucleosomal core and include acetylation, methylation, and ubiquitination (Huang et al., 2014). The dynamics of these histone marks play essential roles in modulating various mRNAs (Gong and Miller, 2013; Lu et al., 2015; Zhou et al., 2020, 2021). Histone acetylation is an essential process during epigenetic modulation in biological processes (Shen et al., 2015). Acetyl groups are dynamically regulated by HATs and removed by HDACs (Chen et al., 2020). At the tails of mainly H3 and H4 of the core histones, histone acetyltransferases (HATs) and Histone Deacetylases (HDACs) are responsible for the dynamics of acetyl-CoA at certain lysine residues. Given that the positive charge on lysine residues is neutralized by acetylated histones, the chromatin structure is loosened. This process weakens the binding of histones, which consequently results in the promotion of transcriptional activity (Eberharter and Becker, 2002). Generally, histone acetylation mediated by HATs is associated with transcription; however, this process has not been fully elucidated in plants (Chen and Tian, 2007), especially in medicinal plants. In detail, HATs have four classes: HACs, which are responsive to the element-binding protein family; TATA-binding protein-associated factor family (HAFs), which are associated with the TATA-binding protein-associated gene family; and N-terminal acetyltransferase family (*HAG*s) and MOZ, Ybf2/Sas3, Sas2, and Tip60 family, MYST (*HAM*s), which contain an acetyltransferases domain (Liu et al., 2016). In addition, HATs have been characterized from several model plants, such as *Arabidopsis thaliana* (Pandey et al., 2002), *Oryza sativa* (Liu et al., 2012), *Litchi chinensis* (Peng et al., 2017), *Hordeum vulgare L* (Papaefthimiou et al., 2010), *Vitis vinifera* (Aquea et al., 2010), *Solanum lycopersicum* (Aiese Cigliano et al., 2013), *Triticum aestivum* (Gao et al., 2021), *Citrus sinensis* (citrus) (Shu et al., 2021), *Setaria italica* (Xing et al., 2022), *Capsicum annuum* (Cai et al., 2022), and *Beta vulgaris L* (Yolcu et al., 2024). The regulatory program of HATs is mediated by dynamic histone acetylation, which is likely related to plant adaptive responses and growth (Shen et al., 2015).

AtGCN5 complexes are known to be associated with cell differentiation and other processes (Vlachonasios et al., 2003). Essentially, in the maintenance of the root stem cell niche, *AtGCN5* activates the transcription of root stem cell-related Transcriptional Factors (TFs) (Kornet and Scheres, 2009). Similarly, with the recruitment of the OsADA2/OsGCN5 complex, the *WUSCHEL*-related homeobox gene controls the crown root meristem (Zhou et al., 2017). In *AtGCN5* and *AtHAF2* mutant plants, light-responsive mRNA transcription was reduced (Bertrand et al., 2003). In *AtHAC* knockdown plants, flowering phenotypes were repressed (Deng et al., 2007). During the development of males and females, *AtHAM1-* and *AtHAM2*-silenced plants exhibit severe defects (Engstrom et al., 2011). In the control of flowering time, H4K5 acetylation mediated by AtHAM1 and AtHAM2 modulates the transcription of MADS-box genes (Xiao et al., 2013).

*Artemisia annua* is popular for its sweet wormwood or Qinghao. *A. annua* is now used globally as the predominant natural source of the potent antimalarial compound artemisinin (Shen et al., 2018). Artemisinin is an endoperoxide sesquiterpene lactone that is considered an effective antimalarial chemical product. Artemisinin is biosynthesized in the glandular trichomes of *A. annua* for the treatment of *Plasmodium falciparum* (Duffy and Mutabingwa, 2006). Therefore, it is very important to increase the artemisinin content and stress resistance under complex environmental conditions. HATs functionally modulate histone acetylation, which may influence plant adaptive responses and growth (Shen et al., 2015). The complete genome sequence of *A. annua* has been released (Shen et al., 2018). This approach has provided an opportunity to study functional genes, especially epigenetic regulators, as little or no research has been conducted on these genes in medicinal plants.

Herein, we examined the whole genome of *A. annua*, identified and characterized the members of the *AaHAT* gene family and analyzed their relationships in a phylogenetic tree as well as their protein structures, patterns at the transcriptional level, *cis*-acting elements of promoters, subcellular localization, and interactions. In total, a group of *AaHAT* genes that have possible functions in medical plants’ responses to dynamic environments was identified.

## Materials and methods

### Identification of the *AaHAT* sequences

We downloaded data containing sequence IDs, protein sequences, genomic sequences, and conserved domain database (CDD) information from the *A. annua* genome database (the NCBI accession number: PKPP00000000). The characterized *AtHAT* and *OsHAT* sequences were obtained from the Phytozome database (https://phytozome.jgi.doe.gov/pz/portal.html V12.1) to identify all *HAT*s in the *A. annua* genome. Eleven putative AaHATs were characterized via BLASTP searches (E< 10^-10^) against the NCBI database. Next, the hidden Markov model (HMM) profiles of HAG (PF00583), HAC (PF08214), HAF (PF09247), and HAM (PF01853) were downloaded from the Pfam database. We applied the same strategy to search for conserved domains in the 11 AaHATs and ensure that they belonged to the HAT family, and TBtools software was used for visualization. Detailed information on the AaHATs was downloaded in batches from the NCBI. Information on the *AaHAT* gene family, which contains gene IDs, protein IDs, location, CDS and protein length, protein molecular weight (MW), isoelectric point (pI), and numbers of exon and groups is provided in detail (**Supplementary Table S1**).

### Multiple alignment and phylogenetic analysis

Multiple sequences were aligned to AtHAT, OsHAT, and AaHAT, and the protein sequences were analyzed using MEGA7.1 (https://www.megasoftware.net/) to construct an unrooted phylogenetic tree via the neighbor joining method with 1000 bootstrap replicates.

### Analysis of gene and protein structures

The genome annotation of *A. annua* was obtained from the NCBI database. Gene structure analysis was conducted using TBtools-II (https://github.com/CJ-Chen/TBtools/releases) with the input of the *A. annua* GTF file. We used SWISS-MODEL to predict the structures of the AaHATs. The online tool multiple Em for motif elicitation was applied to analyze protein motifs.

### *Cis-*elements in the *AaHAT* promoter

We defined upstream 2Kb sequences counted from the ATG start codon as the promoter regions with the help of TBtools-II. These promoter sequences were then used to identify *cis*-elements in the predicted promoter in the website of PlantCARE (https://bioinformatics.psb.ugent.be/webtools/plantcare/html/). The *cis*-elements were defined and grouped with their various functions. Cis-elements of abscise acid responsiveness (ABRE), gibberellin-responsive elements (P-box elements), and auxin responsive elements (TGA-element) were identified in the promoter regions of the 11 AaHAT.

### Analysis of transcriptional profiles

Data of the transcription pattern of *AaHAT* genes in six tissues of *A.annua* were downloaded from a published NCBI database (https://www.ncbi.nlm.nih.gov/) (SRA accession number: SRP129502). These tissues included young and old leaves, stem, seeds, roots, and flowers. All RNA-seq library preparation was conducted as described in previous study. Heatmap Illustrator HemI 2.0 (http://hemi.biocuckoo.org/) was used to plot the heatmap of gene transcription.

### Plant materials, growth conditions and stress treatments

Plantlets were cultivated in growth chambers under optimal conditions of temperature (25 ± 2°C) and light provided by cool white fluorescent tubes emitting a light intensity of 40 μmol m^−2^ s^−1^. All plantlets were subjected to a 16-hour photoperiod. Mass propagation of plantlets was achieved through sub-culture in MS medium every 15 days. To investigate the responses of *AaHATs* to various abiotic stresses, 2-week-old plants grown on plates with moist gauze were treated with a solution containing 200mM NaCl and 100 μM ABA. For cold treatments, the plants were exposed to 4°C. Leaves from all samples for RNA extraction were collected at 0 h, 1 h, 2 h, 5 h, and 24 h post-treatments.

### RNA isolation and quantitative real-time PCR analyses

Total RNA for quantitative real-time PCR (qRT-PCR) analysis was extracted using Trizol reagent (Invitrogen) following the manufacturer’s protocol. Subsequently, cDNA was generated from 2 μg of total RNA utilizing the EasyScript One-Step gDNA Removal and cDNA Synthesis Supermix (Transgene Biotech, China). The qRT-PCR analysis was conducted with TranStart qPCR SupMix chip (Transgene Biotech, China) on the ABI StepOnePlus real-time PCR system with a minimum of three biological replicates. Data analysis was performed using the comparative Ct (ΔΔCt) method in Excel. Statistical significance between treatment and control groups was determined using Student’s t-test, with *p* values below 0.05 or 0.01 considered significant.

## Results

### Identification of *HAT* genes in *A. annua* genome

**Figure 1:**
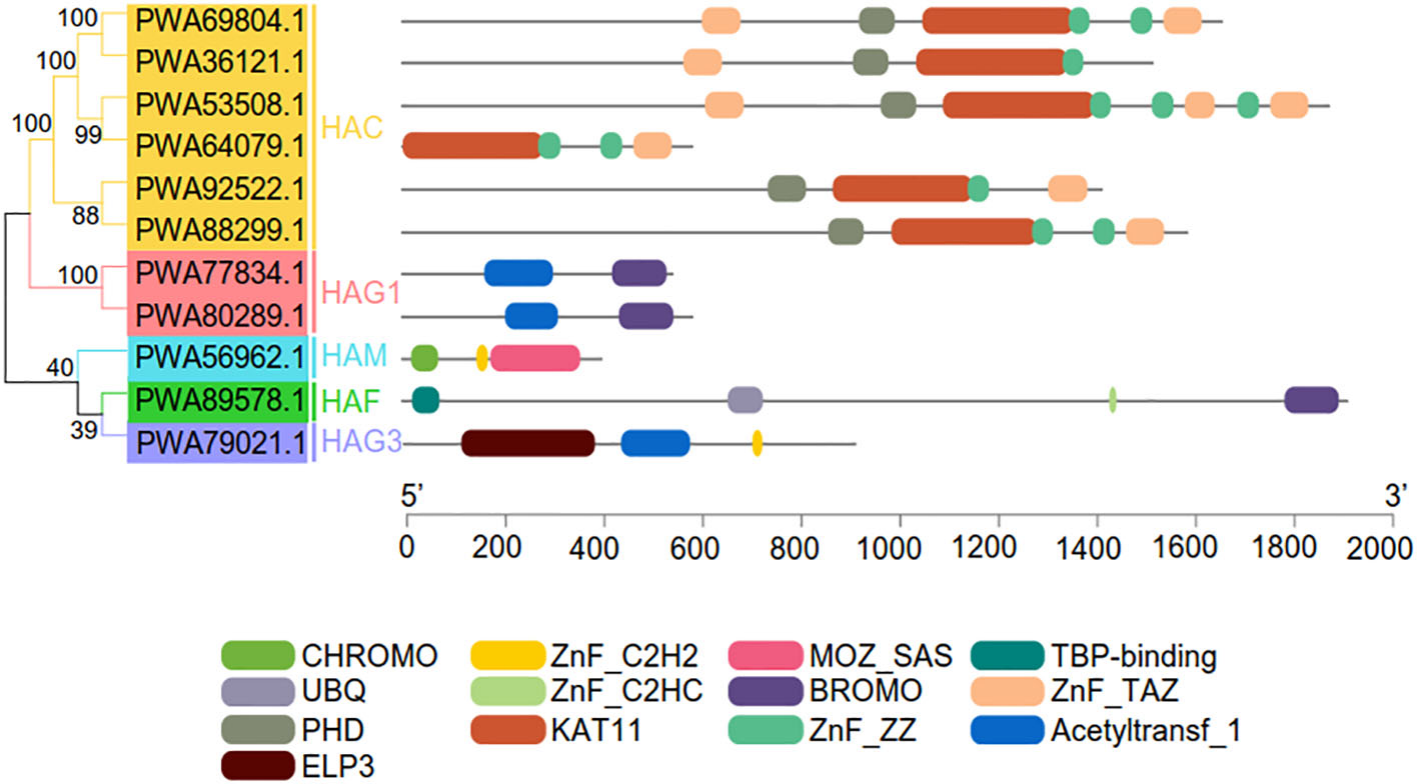
Conserved domain analysis of the *AaHAT* gene family. The 11 *AaHAT* can be divided into six groups (HAC, HAM, HAF, HAG1, HAG2, and HAG3) based on conserved domain analysis. Individual conserved domains are indicated by different colored boxes.

We identified and characterized 11 HATs in *A. annua* with *A. thaliana* and rice HATs as queries (**Supplementary Table S2**). Consistent with their evolutionarily conserved sequences and the identified HATs in *A. thaliana*, the 11 AaHATs were classified into five groups with short names: HAC, HAG1, HAG3, HAF, and HAM. Every group had individual conserved domains, which reinforced the relevance of the classification (**Figure 1**). Approximately 60% of the AaHAC CDSs were 800–2000 aa long, while PWA56962.1 (385 aa), PWA64079.1 (561 aa), PWA77834.1 (521 aa), and PWA80289.1 (561 aa) were unique. The protein sequences of each group displayed substantial similarity. The molecular weights of the AaHATs ranged from 45.31 to 207.58 kDa. The isoelectric points varied from 5.188 to 8.443. PWA89578.1 encoded the largest protein with a molecular weight of 207.58 kDa, but PWA56962.1 encoded the smallest protein with a molecular weight of 45.31 kDa (**Supplementary Table S1**). The protein nature of the AaHATs is highly similar to that of HATs from other plants (Pandey et al., 2002; Liu et al., 2012), which indicates that the functions of these AaHATs are evolutionarily conserved.

### Phylogenetic analysis of the *AaHAT* proteins

**Figure 2:**
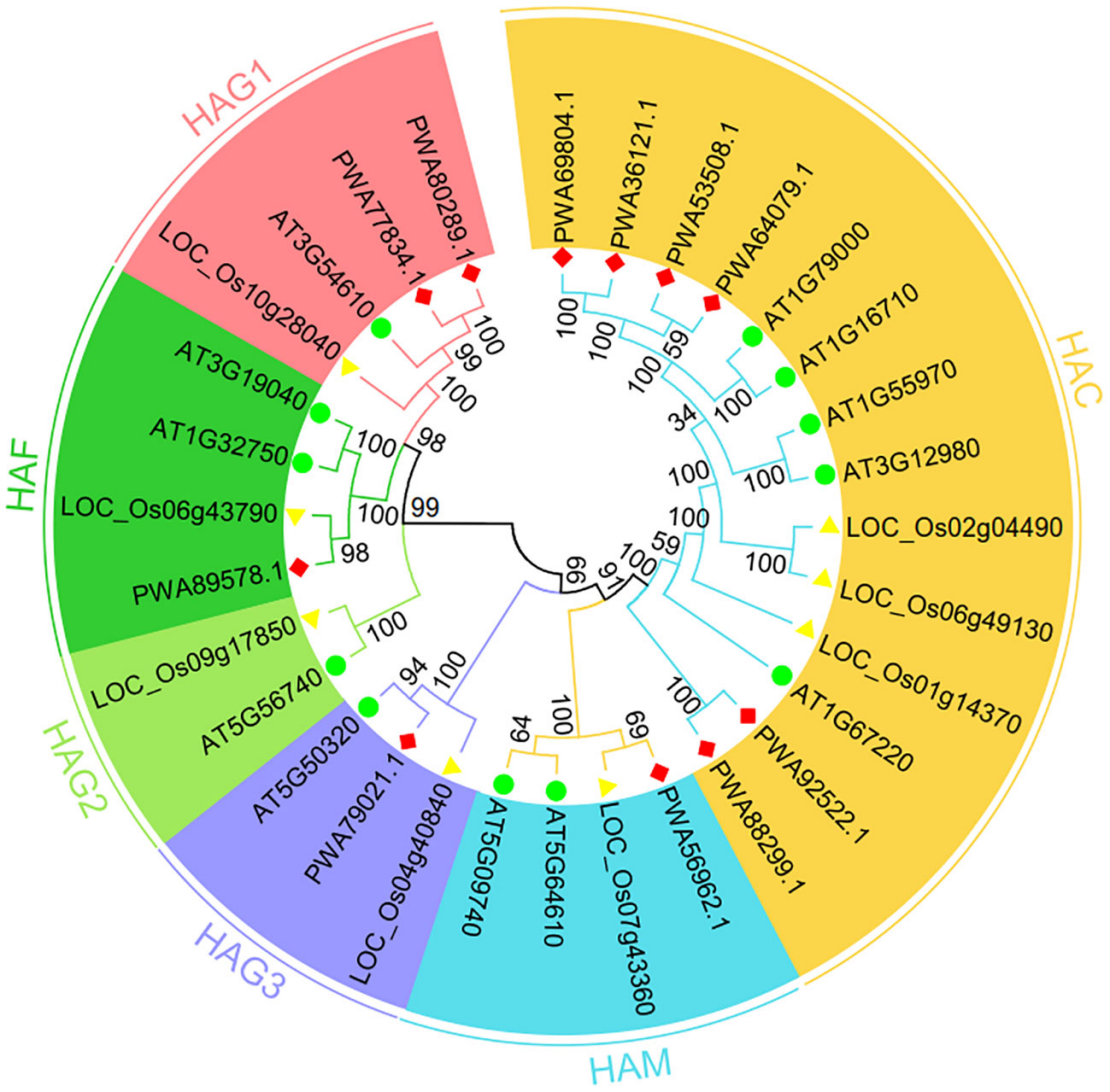
Phylogenetic tree of HAT proteins from *Arabidopsis thaliana*, *Oryza sativa* and *Artemisia annua* constructed by the neighbor-joining method in MEGA-X. The numbers at nodes represent bootstrap values after 1000 iterations. Each group is indicated by a different color. Circles represent *A. thaliana*, triangles represents *O. sativa*, and rhombus represent *A. annua*.

To probe the phylogenetic relationships of plant AaHATs, we generated twelve *A. thaliana*, eight *O. sativa*, and eleven *A. annua* HAT amino acid sequences which were used to construct a neighbor joining tree. There were no assumptions about ancestral representation based only on the relationships of the leaf nodes in unrooted trees. As a consequence, HATs from the three plant species, *A. thaliana*, rice, and *A. annua*, were divided into six groups, as expected (**Figure 2**). The AaHAT proteins exhibited strong similarity with HATs from module plants. These genes were classified into clades with AtHATs and OsHATs, which had better bootstrap values. One AtHAT, one OsHAT, and two AaHATs were grouped into the HAG1 clade, while for the HAG3 group, each species had one HAT (**Figure 2**). Intriguingly, there was no AaHAT in the HAG2 group (**Figure 2**). These data indicated that the HAT in the *A. annua* genome has unique biological evolution compared with that of these module plants. Regardless of the plant species, the HAC group had the largest group, with five AtHATs, three OsHATs, and six AaHATs (**Figure 2**). Two AtHATs and OsHATs were clustered into HAM and HAF (**Figure 2**). Overall, we found that HATs in the *A. annua* genome are highly conserved in terms of evolution and are rich in highly variable regions; thus, HATs have become comparatively ideal objects of medical plant identification and systematic study.

### Predicted structure and conserved motifs analysis of *AaHAT* proteins

To visualize the various protein structures of histone acetylation transferases, we randomly selected HATs from each group of three plant species for analysis (**Supplementary Figure S1**). HAM proteins have similar structures among diverse plant species (**Supplementary Figure S1**). In contrast, the HAC, HAF, and HAG protein structures appeared to diverge from each other in this study (**Supplementary Figure S1**). In addition, we found that the conserved sequence of the HAT protein was complete, but only partial folding regions differed slightly (**Supplementary Figure S1**). *AaHACs* had one special gene (*PWA64079.1*) that evolutionarily lost regional introns. Consequently, the 3D structures of these genes are similar to those of other family groupers of AaHAC (**Figure 1** and **Supplementary Figure S2**). In total, for the same groups of HATs, we found that these proteins from different plant species possess similar structures, and vice versa. Given that protein structure and character determine the properties and functions of organisms, these data suggest that HATs from these three plant species may have homologous biological functions.

**Figure 3:**
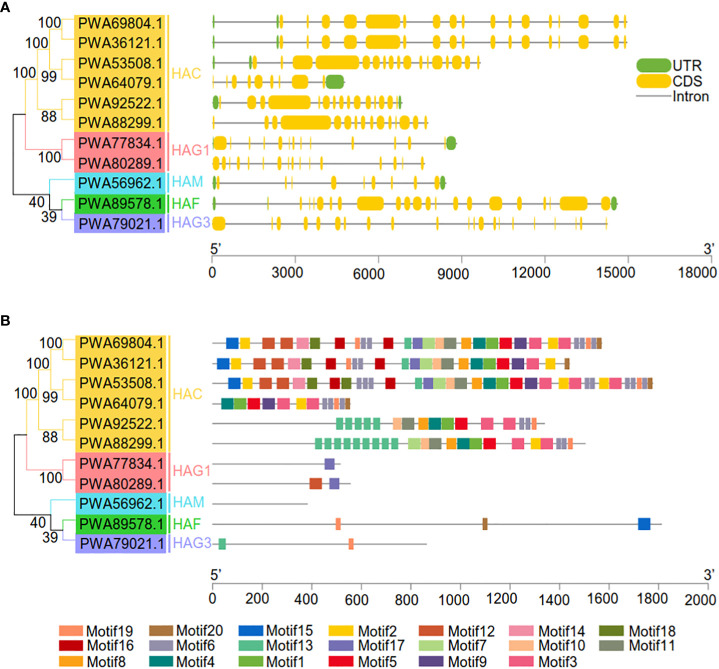
Genetic and motif structures of 11 *AaHAT* genes. **(A)** Exons, introns, and untranslated regions (UTR) are indicated by yellow frames, gray lines, and green frames on the right, respectively. The number on the gray line represents the number of introns. **(B)** Schematic representation of twenty conserved motifs in the AaHATs. Different colored frames represents different protein motifs, and each motif has its own number.

To better understand gene family evolution, we compared the structures of the AaHATs (**Figure 3A**). Analysis of the genomic DNA sequences revealed that the number of introns varied from 8 to 20 (**Figure 3A**). *AaHAT*s mostly shared highly similar structures, which were grouped together in three branches of the NJ tree (**Figure 3A**). The majority of the *AaHAC*s had similar numbers of introns and exons, with the exception of one *AaHAC* gene (PWA64079.1). *AaHAF* and *AaHAG3* had the greatest number of introns (20) among all the groups. Moreover, to identify putative motifs in the *HAT* family in *A. annua*, we predicted the protein sequences of the 11 AaHATs using the MEME website. Consequently, 20 motifs were identified from these proteins (**Figure 3B**; **Supplementary Table S3**). The group contained similar motifs, which indicated that the proteins might have some common functions. *HAC* genes are evolutionarily conserved in other plants and possessed the greatest number of motifs. There was at least one motif in each AaHAT, and they were arranged in the same order in each group except for AaHAM (PWA56962.1), which had no predicted motif. Overall, this analysis supported the presumption that motifs commonly present in histone acetyltransferases in the *A. annua* genome may be related to the conserved functions of *AaHAT*s, whereas the *AaHAT*s seem to be associated with specific functional rules.

### Subcellular localization prediction of *AaHAT*

Given that subcellular location details can provide some clues for the prediction of protein function, we found that all of the AaHATs were predicted to be localized in the nucleus with high reliability (RI > 6), except for HAM (PWA56962.1), which might be localized in the chloroplast, mitochondria, and nucleus (**Supplementary Table S4**). In particular, HAC (PWA69804.1) was located only in the nucleus with high reliability (RI = 14), and other AaHATs were predicted to be located in at least two subcellular organelles (**Supplementary Table S4**). Similarly, Uniprot identified possible nuclear signals for AaHATs, except for HAM (PWA56962.1) and HAG3 (PWA79021.1), which were not predicted. The identified AaHAT proteins in the *A. annua* genome exhibited different subcellular distributions and may be involved in medical plant developmental growth.

### Spatial and temporal expression of *AaHATs* in various developmental tissues of *A. annua*

**Figure 4:**
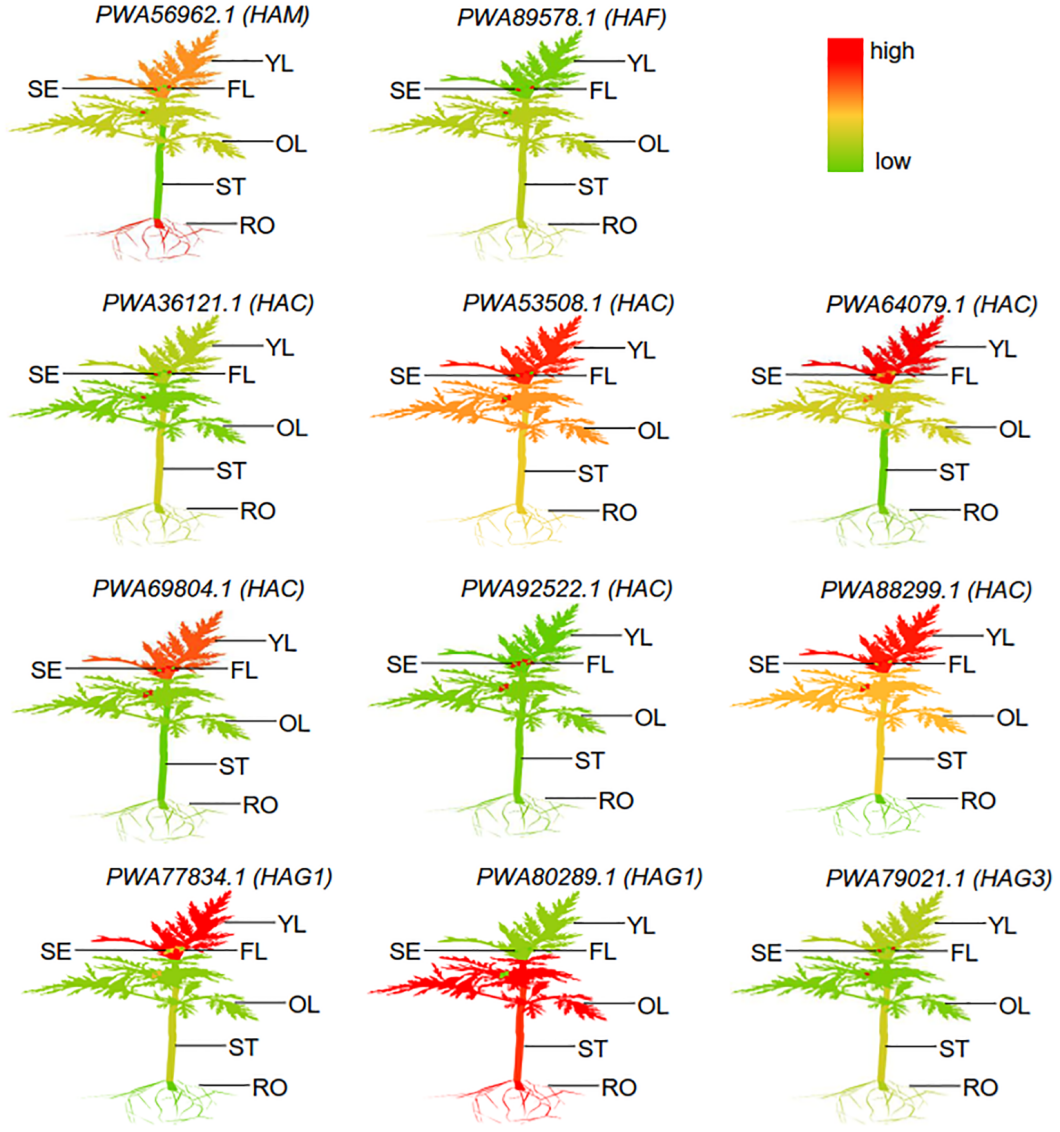
Different expression of representative *AaHATs* in different tissues. YL, Young-leaf; FL, Flower; SE, Seed; OL, Old-leaf; ST, Stem; RO, root. The mean expression values were visualized by Tbtools; red represents a high expression level and green represents a low expression level. These RNA-seq data different tissues was download from public database (**Supplementary Table S4**).

To determine the transcriptional levels of the *AaHAT* genes in different organs, we generated a heatmap with the RNA-seq data from the *A. annua* database (**Supplementary Table S4**). We detected complex, specific, and overlapping *AaHAT*s in different tissues. The tissues/organs were divided into six types: flowers, young leaves, old leaves, seeds, stems, and roots. According to the results, 11 *AaHAT* genes were distinctly expressed at different developmental stages in the tissues. The transcriptional level of the genes was dynamic among these tissues and organs. For example, *PWA92522.1 (HAC)* was barely detectable in whole plants; in contrast, transcriptional signals of the *PWA53508.1 (HAC)* gene were highly detected in the overall plantlet (**Figure 4**). Additionally, the transcript levels varied within the same organs. In the six *A. annua* tissues, the expression of the members of the AaHAC gene family was relatively low (**Figure 4**). Some genes were exclusively transcribed in single tissues or organs; for example, *PWA69804.1 (HAC)* had a high transcriptional level in old leaves. The transcriptional levels of the *AaHATs* varied among tissues and were involved in the development of *A. annua*.

### Prediction of *cis*-acting elements in the promoter regions of the *AaHAT* genes

**Figure 5:**
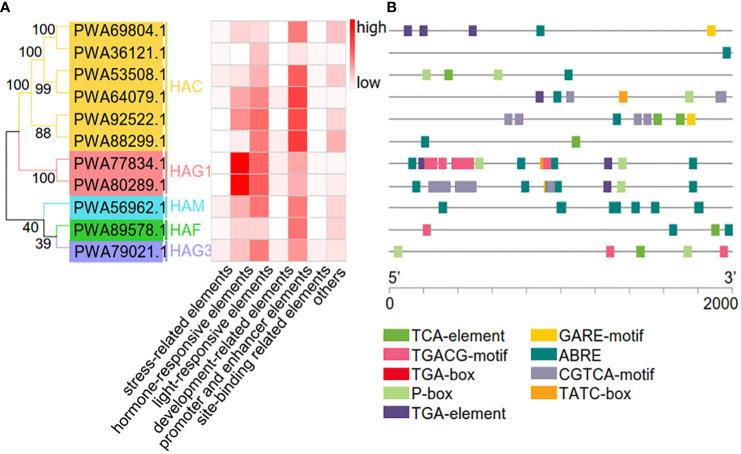
Prediction of cis-acting elemets in the *AaHAT* promoters. **(A)** Number of *cis*-acting elements detected in the promoter region of each *AaHAT* gene; elements were divided into seven types. **(B)** Type, quantity, and position of hormone-responsive elements in *AaHATs*’ promoters. MeJA-responsiveness (TGACG-motif, CGTCA-motif), auxin-responsive (TGA-box, TGA-element), abscisic acid responsiveness (ABRE, TCA-element), gibberellin-responsive (GARE-motif, P-box, TATC-box).

To better understand the potential roles of *AaHATs*, we used a plant promoter database to identify the region 2 kb upstream of the transcription initiation site of *AaHATs*. Overall, we identified 619 *cis*-acting elements within the promoter regions of the *AaHAT*s. The associated elements included stress-related, hormone-responsive, light-responsive, developmental, promoter and enhancer, site-binding-related, and other elements (**Figure 5A**). Compared with those of other elements, the abundance of light-responsive and promoter and enhancer elements increased (**Figure 5A**). In addition, *cis*-elements in the promoter regions of the AaHATs were found to be relevant to plant hormone classifications (**Figure 5B**). As a consequence, all 11 AaHATs contained abscisic acid responsive-elements (ABREs), while gibberellin-responsive (P-box elements) and auxin responsive (TGA-element) elements were identified in practically all *A. annua* HAT promoters (**Figure 5B**). Interestingly, obvious *cis*-elements between the paired genes, including gibberellin and MeJA response elements, were identified in the promoter of *AaHAG1* (*PWA77834.1*) (**Figure 5B**). Notably, no cytokine-responsive elements were found in these promoters (**Figure 5B**), which was consistent with previous results (Xing et al., 2022). These results showed that *AaHAT*s may affect phytohormone responsiveness, adaptability to stress, and developmental growth.

### Regulatory network mediated by *A. annua* HAT genes

It is important to study gene function to construct gene family interaction networks. Here, we constructed a regulatory network controlled by *A. annua* HAT genes with STRING. Among the 11 AaHATs, 9 were identified to interact with proteins with high confidence (score > 0.8). AaHAC, which has four groups, namely PWA88299.1, PWA53508.1, PWA88299.1, and PWA64079.1; AaHAG1, which includes PWA80289.1 and PWA77834.1; PWA89578.1 (HAF); PWA56962.1 (HAM); and PWA79021.1 (HAG3) constitute an independent regulatory network with 34 proteins involved in crosstalk (**Supplementary Table S6**).

In detail, we determined the PCC values corresponding to the *A. annua* transcriptome of dynamic organs in the network. In our analysis, the red rectangles represent AaHATs, and the red edges represent the PCC values of AaHATs and interaction scores > 0.5 (**Supplementary Figure S3**). Among the nine AaHATs, nine expressed transcripts had PCCs > 0.5 for PWA88299.1 (AaHAC), which was highly expressed in young flowers and young leaves; eight genes had PCC values greater than 0.5 for PWA53508.1 (AaHAC), which was most highly expressed in young/old leaves (**Supplementary Figure S3**). Five genes had PCC values > 0.5 for PWA64079.1 (AaHAC), which was preferentially expressed in young leaves, and 2 genes had PCCs > 0.5 for PWA88299.1 (AaHAC), which also exhibited high transcription in young leaves (**Supplementary Figure S3**). Four genes had PCC values greater than 0.5 for PWA77834.1 (AaHAG1), and these genes were highly expressed in young leaves (**Supplementary Figure S2**). The 3 genes had PCCs >0.5 for PWA80289.1 (AaHAG1), which was most highly expressed in old leaves, stems, and roots, and 2 genes had PCC values > 0.5 for PWA79021.1 (HAG3), which was preferentially expressed in seeds (**Supplementary Figure S3**). Four genes had PCC values > 0.5 for PWA89578.1 (AaHAF) and were highly expressed in the seeds (**Supplementary Figure S3**). The four genes had PCCs > 0.5 for PWA56962.1 (AaHAM) and were highly expressed in young leaves and roots (**Supplementary Figure S3**). Interestingly, *PWA94397.1*, which is similar to the Arabidopsis and rice *ARBE* (abscisic acid responsive element) binding factors, was one of the genes expressed with *AaHAG1* (*PWA80289.1*), with a PCC greater than 0.5 (**Supplementary Figure S3**; **Supplementary Table S6**). Furthermore, the entire promoter of the predicted AaHATs contained ABRE *cis*-elements (**Figure 5**). Thus, we speculate that AaHAG1 may interact with other binding factors of the AaHAT gene, which indicates that HATs in *A. annua* may have self-regulating systems.

### Roles of *AaHAT* genes in the response of *A. annua* to stress

**Figure 6:**
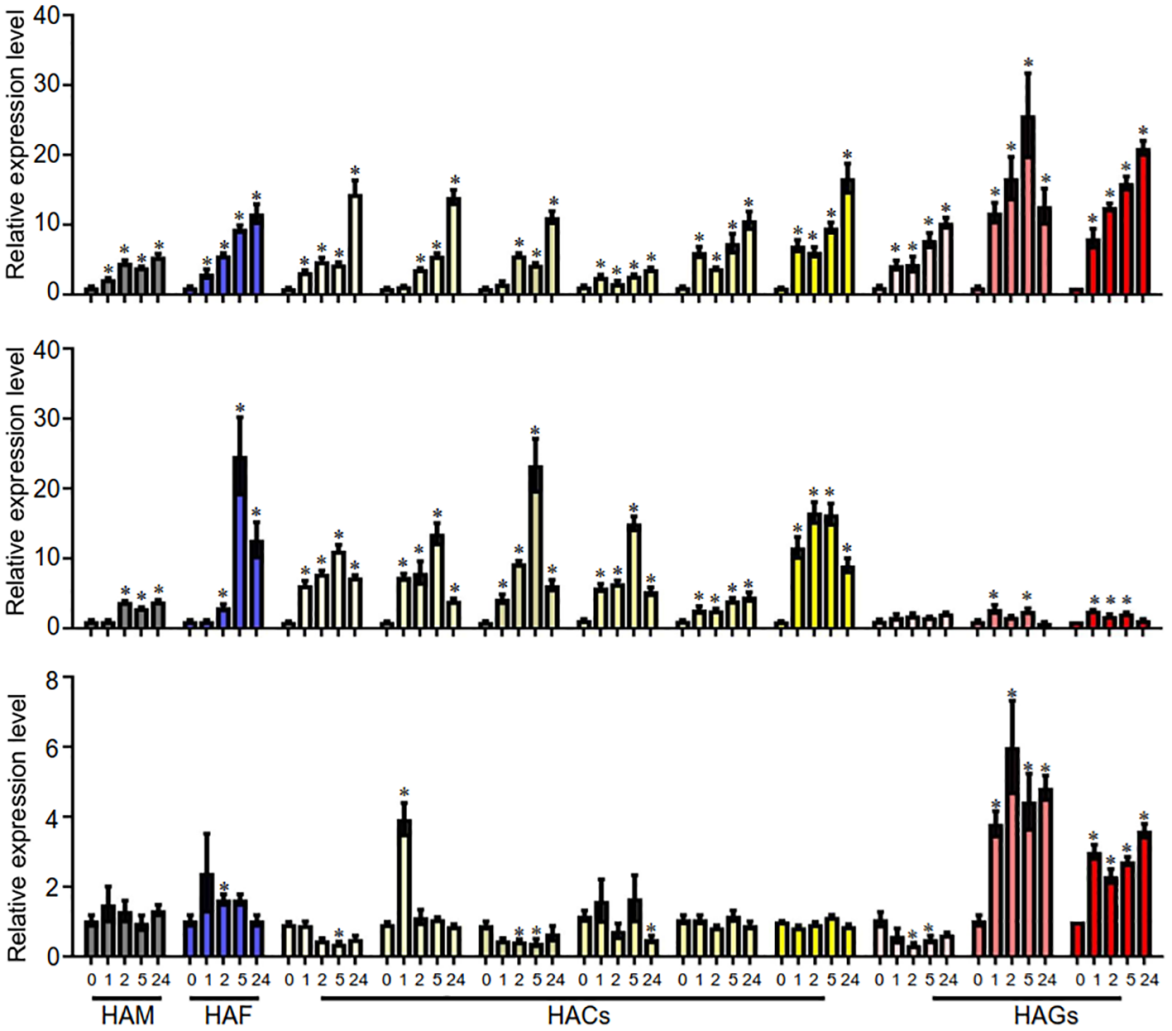
qRT-PCR analyses of *AaHAT* genes under the treatments of three different abiotic stresses. The transcript levels of *AsHAT* gene under cold, NaCl and ABA treatments and mock were normalized. The average ± SD values from three biological repeats are shown. *P < 0.05, Student’s t-test.

HATs may be involved in plant responses to environmental stresses. In this study, we investigated the expression levels of *AaHATs* under three treatments, cold, NaCl, and ABA, via qRT-PCR. We found that the expression of the AaHAT genes increased at most cold treatment time points. In addition, *AaHACs* were induced quickly with NaCl treatment and decreased after 5 hours of NaCl exposure in comparison with those in the control group (**Figure 6**). Interestingly, the expression of orthologs of these genes in rice increased after NaCl treatment, which implies the evolutionary conservation of HAT genes in the plant kingdom. Interestingly, the expression profiles of the AaHAC proteins exhibited obvious positive correlations with all PCCs > 0.6 in both our tested organs and stress treatments, suggesting functional redundancy of the AaHAC proteins in *A. annua* (**Supplementary Figure S3**). In addition, we also found that some associated genes in the artemisinin biosynthetic pathway transcriptionally activate under stress. In detail, in terms of cold stress, the expression levels of the four artemisinin biosynthetic genes were also significantly increased, such as *AaADS*, *AaCYP71AV1*, and *AaDBR2* (**Supplementary Figure S4A**). As for NaCl treatment, the expression of AaADS and AaDBR2 were induced (**Supplementary Figure S4B**). After ABA application, we found that only AaCYP71AV1 was active (**Supplementary Figure S4C**). The results reinforced the assumption that the HAC proteins in monocot plants have shared ancestral genes, which may activate artemisinin biosynthesis related genes by ectopic acetylation under cold and NaCl treatment. However, it was not obvious that *AaHATs* were induced under ABA treatment, including the downstream genes (**Figure 6**). In summary, it was indicated various *AaHATs* had special roles under various environment stimuli, which may activate artemisinin biosynthesis associated genes to promote the plant-derived output.

## Discussion

The artemisinin component from *A. annua*, an important traditional Chinese medicine, is the most effective drug against malaria (Rolta et al., 2021). The *HAT* family generally consists of complex groups and plays a very important regulatory role in numerous cellular processes (Kikuchi and Nakayama, 2008; Mai et al., 2009). Consequently, the activities of HAT proteins are associated with plant developmental growth and stress responses (Wang et al., 2014). Thus, the identification and characterization of *HAT* gene families have provided an opportunity to study functional epigenetic regulators responsive to environmental dynamics in medical plants. Herein, we characterized 11 *HAT*s in the *A. annua* genome and investigated their phylogenetic relationships, protein structures, transcriptional patterns, *cis*-acting elements of promoters, subcellular localization prediction, and interactions. Furthermore, we identified the *AaHAG*s as *HAG1* and *HAG3* according to the dynamics of their conserved domains, which evolutionarily lost the *HAG2* group (**Figure 1**). The protein properties of the AaHATs are highly similar to those of HATs from other plants (Pandey et al., 2002; Liu et al., 2012), which indicates that the functions of these AaHATs are evolutionarily conserved. Together, we found that HAT in *A. annua* genome has high conservatism of evolution and at the same time is rich in highly variable regions, and thus has become a comparatively ideal object of medical plants identification and systematics study.

Next, we constructed neighbor-joining trees to display the full-length protein sequences of HATs in *A. annua*, *A. thaliana*, and *O. sativa*. As expected, the neighbor joining tree was divided into six clades (**Figure 2**). Regardless of the plant species, the HAC group possessed the largest group, with 5 AtHATs, 3 OsHATs, and 6 AaHATs (**Figure 2**). Moreover, since the structure of proteins impacts the function of genes, our analyzed data indicated that HATs from three plant species may perform homologous biological functions (**Supplementary Figure S1**). In *Arabidopsis*, three AtHAC genes play vital roles in the control of flower development by repressing the transcription of *FLC* (Deng et al., 2007). Thus, the results indicated that AaHAC, which is homologous to AtHACs, may therefore be involved in *A. annua* flowering (**Figure 2**). According to phylogenetic trees of their protein sequences, AaHAFs shared similar functional rules with AtHAF1 (**Figure 2**). AtHAF1 knockdown plants were able to adapt to plant transformation mediated by *Agrobacterium* (Crane and Gelvin, 2007). AtHAF2 modulates the transcription of several cold-responsive genes (Pavangadkar et al., 2010). The AaHAM proteins shared high similarity with HAM proteins from module plants. Given that AtHAM participates in the development of males and females (Nelissen et al., 2005), it was hypothesized that the closest AaHAM orthologous genes may possess similar functions. Intriguingly, no AaHATs existed in the HAG2 group (**Figure 2**), which indicated that HAT in the *A. annua* genome has unique biological evolution compared with that of these module plants. In addition to HAG2, AaHAG1 and HAG3 share similarities with AtHAG1 and AtHAG3 (**Figure 2**). *AtHAG1* plays vital roles in the developmental stages of cell differentiation. *AaHAG1* (*PWA77834.1*) is highly expressed in flowers (**Figure 4**) and may have similar functional patterns to AtHAG1 (**Figure 2**), which indicates that AaHAG1 (*PWA77834.1*) may play a role in flower development. Additionally, most of the AaHATs were predicted to be localized in the cell nucleus with high reliability (**Supplementary Table S4**), which indicated that these AaHATs may modulate epigenetic marks in the cell nucleus and result in dynamic transcriptional activities. Similarly, AtHAG3 can also modulate the plant response to ABA (Chen et al., 2006), which is consistent with the finding of abscisic acid responsiveness (TCA-element) in the promoter of *AaHAG3* (**Figure 5**). Overall, we found that HATs in the *A. annua* genome, which are highly conserved throughout evolution, have become comparatively ideal objects of medical plant identification and systematic study.

Increasing evidence for the dynamics among multiple-copy genes could be associated with transcriptional trends for multiple AaHAT gene sets. *PWA92522.1 (HAC)* was barely expressed in the whole plant; however, the transcriptional level of *PWA53508.1 (HAC)* was high in the overall plantlet (**Figure 4**). According to the results, 11 *AaHAT* genes were distinctly expressed at different developmental stages in the tissues. In addition, a possible function was clearly observed in the transcriptional patterns. For instance, *PWA69804.1 (HAC)* was highly expressed in old leaves; in contrast, the mRNA of *PWA92522.1* (*HAC*) was highly expressed in seeds (**Figure 4**), which agreed with previous results (Wang et al., 2014). Additionally, AtHAG1/GCN5 is a well-known histone acetyltransferase that modulates the development of the leaf and floral meristem (Servet et al., 2010). Among these genes, *PWA77834.1* (*HAG1*) exhibited a similar pattern of high expression in flowers and leaves (**Figure 4**). In rice, the ADA2-GCN5 complex recruits WOX11, which controls the crown root meristem (Zhou et al., 2017). Similarly, *PWA80289.1* (*HAG1*) was highly transcribed in roots and may play vital roles in root development (**Figure 4**).

The *cis*-acting elements are associated with a dynamic environment (Li et al., 2019). We found that 619 *cis*-elements were located in the promoters of 11 *AaHAT*s. The elements were associated with stress-related, hormone-responsive, light-responsive, developmental, promoter, site-binding-related, and other elements (**Figure 5A**). All 11 *AaHAT*s contained ABREs, while P-box elements and TGA elements were identified in practically all *A. annua* HAT promoters (**Figure 5**). Notably, no cytokinin-responsive elements were identified in these promoter regions (**Figure 5**), which was consistent with previous results (Xing et al., 2022). We also investigated the expression levels of these *AaHAT*s under cold, NaCl, and ABA treatments (**Figure 6**). These results showed that *AaHAT*s may affect phytohormone responsiveness, adaptability to environmental stress, and developmental growth. Interestingly, the transcriptional levels of four *AaHACs* (**Supplementary Figure S3**) had obvious positive correlations in the tissues, which indicated the functions of the four AaHACs in *A. annua*. In accordance with our assumption, the AaHACs in monocots were assumed to have the same ancestral genes.

Notably, these adversity stresses could enhance the production of artemisinin to different extents (Paul and Shakya, 2013; Ji et al., 2014; Liu et al., 2017). Research indicated that artemisinin biosynthesis could be positively promoted by various environmental factors and was tightly regulated by spatial and temporal transcripts in the biosynthetic pathway. This regulation was genetically controlled by different types of transcriptional factors, such as *AabHLH* and *AaERF* (Paul and Shakya, 2013; Ji et al., 2014). In addition to transcriptional factors, epigenetic regulation was also considered an important molecular mechanism for natural plant-derived products under environmental stresses (Abdulraheem et al., 2024). Furthermore, our results indicated those AaHAT genes and some key genes in the artemisinin biosynthetic pathway were active under cold and NaCl stress (**Figure 6**; **Supplementary Figure S4**). It was hypothesized that histone acetyltransferases may interact with transcription factor proteins to regulate downstream genes. For example, the transcriptional coactivator AtADA2b, which is the homolog of the yeast ADA2 protein, has been demonstrated to physically interact with AtGCN5 and the cold-induced transcription factor AtCBF1, a member of the C-repeat/DRE-binding factors (Mao et al., 2006). Therefore, how epiregulatory factors and transcription factors promote the production of medicinal ingredients when medicinal plants respond to environmental stress is a project worthy of in-depth study.

## Conclusion

*A. annua*, which is considered to be the only source of artemisinin, the most effective therapeutic drug for malaria, is sensitive to the surrounding growing habitat. HAT activity controls chromatin modification and mRNA transcription, resulting in the regulation of plant adaptation to dynamic environments. Thus, a systematic study of *AaHATs* during *A. annua* development provides insight into the relationship between HATs and environmental adaptation in medicinal plants. Here, we examined the whole genome of *A. annua*, identified and characterized the members of the *AaHAT* gene family, and constructed phylogenetic trees of the members of the AaHAT gene family in comparison with those of model plants. We analyzed the resulting gene structures, expression profiles, *cis*-acting elements of promoters, subcellular localization predictions, and interactions. Overall, a set of *AaHAT* genes that have potential roles in the response to environmental changes was identified in this study.

## Data availability statement

The original contributions presented in the study are included in the article/**Supplementary Material**. Further inquiries can be directed to the corresponding author.

## Author contributions

YG: Data curation, Funding acquisition, Writing – original draft. YY: Formal analysis, Investigation, Software, Writing – review & editing. FC: Formal analysis, Software, Visualization, Writing – review & editing. YL: Writing – review & editing.
