# Supplementary material for: Identification of the histone acetyltransferase gene family in the Artemisia annua genome
Source: Front Plant Sci. 2024 Jul 24;15:1389958. doi: 10.3389/fpls.2024.1389958 (PMC11303224; doi:10.3389/fpls.2024.1389958)
Supplement: Supplementary file 1 [file Image_1.pdf]

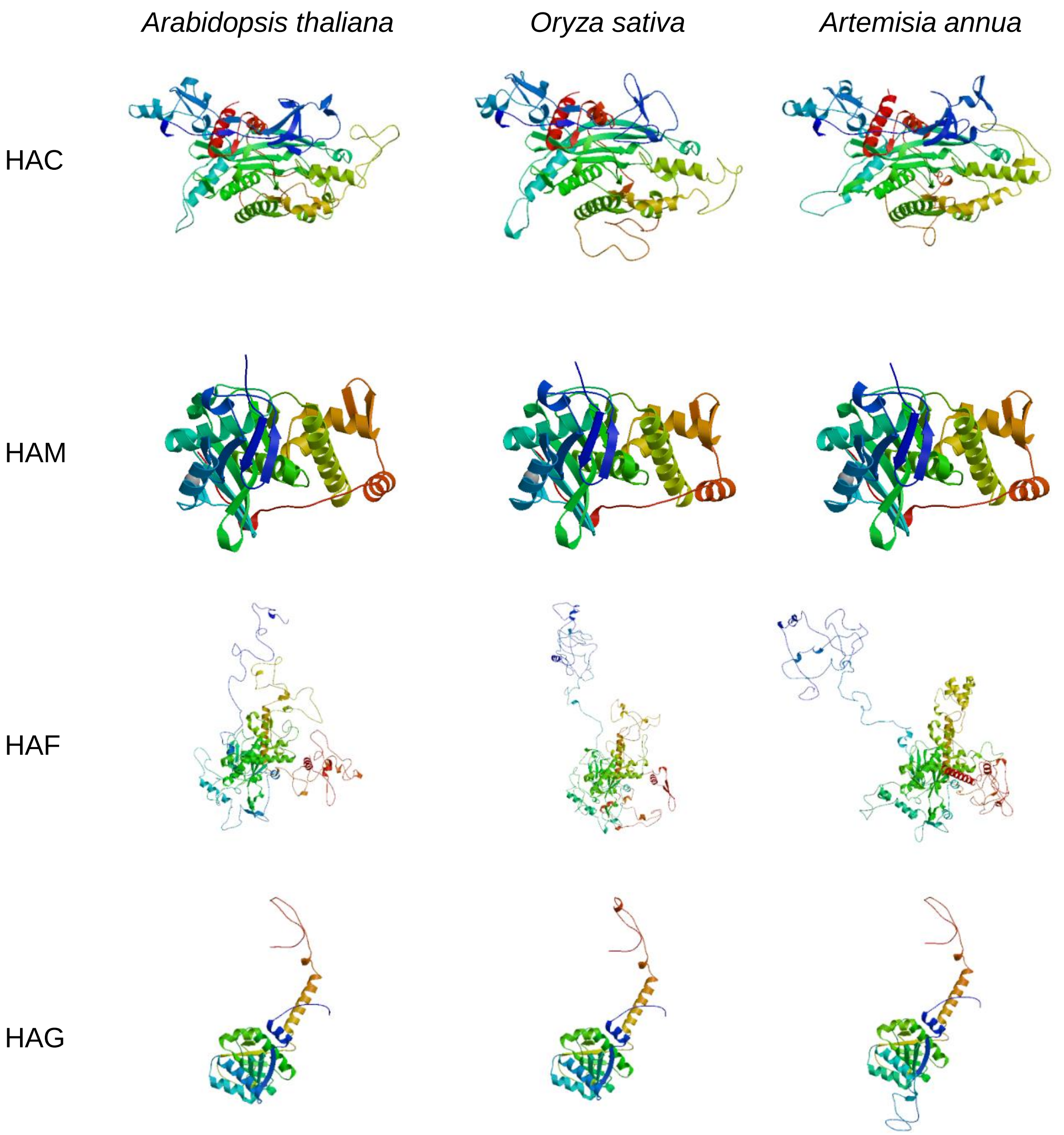

Fig. S1 Predicted structures of AaHATs proteins.
A gene model display is randomly selected from each group: HAC (AT1G79000, LOC_Os01g14370, PWA53508.1), HAM (AT5G64610, LOC_Os07g43360, PWA56962.1), HAF (AT1G32750, LOC_Os06g43790, PWA89578.1), HAG (AT3G54610, LOC_Os10g28040, PWA80289.1).

AaHAC *PWA64079.1*

HAC

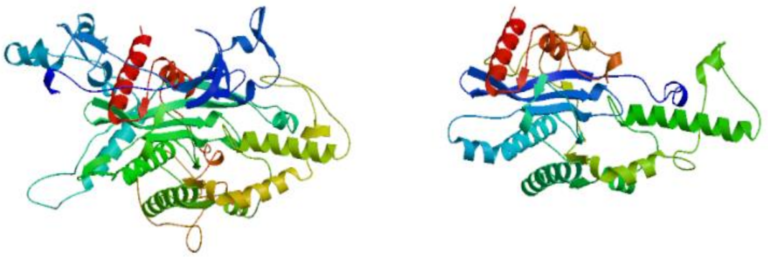

Fig. S2 Predicted structures of one AaHAC protein.

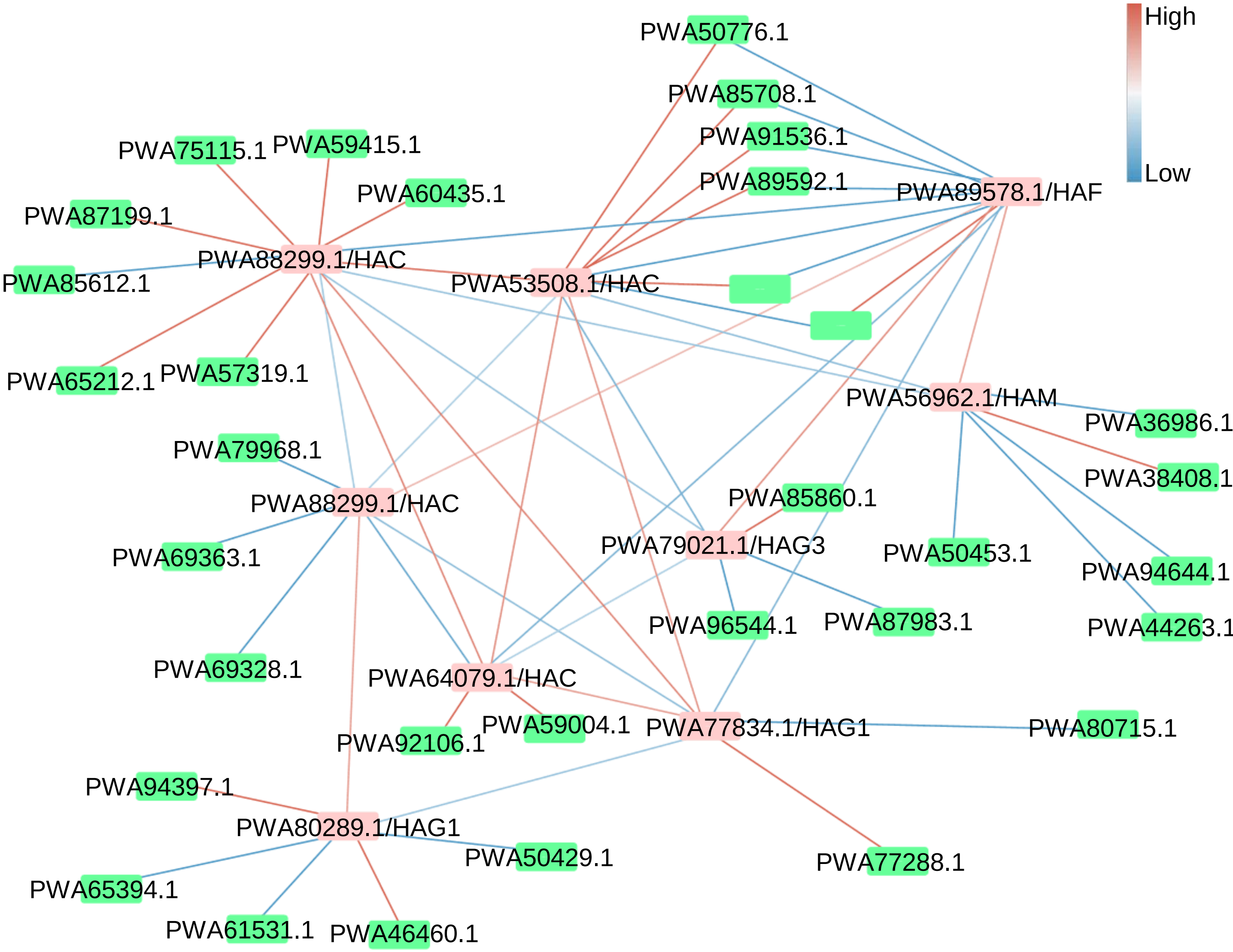

Fig. S3 Interaction network and co-expression analyses of nine AaHAT genes at different developmental stages of *A. annua*.

The red rectangles (nodes) represent AaHATs. The AaHATs and its interaction targets are displayed when their PCCs of expression profiles are larger than 0.5 (For interpretation of the references to colour in this figure legend, the reader is referred to the web version of this article). Red represent high interaction and blue represent low interaction between two candidated proteins.

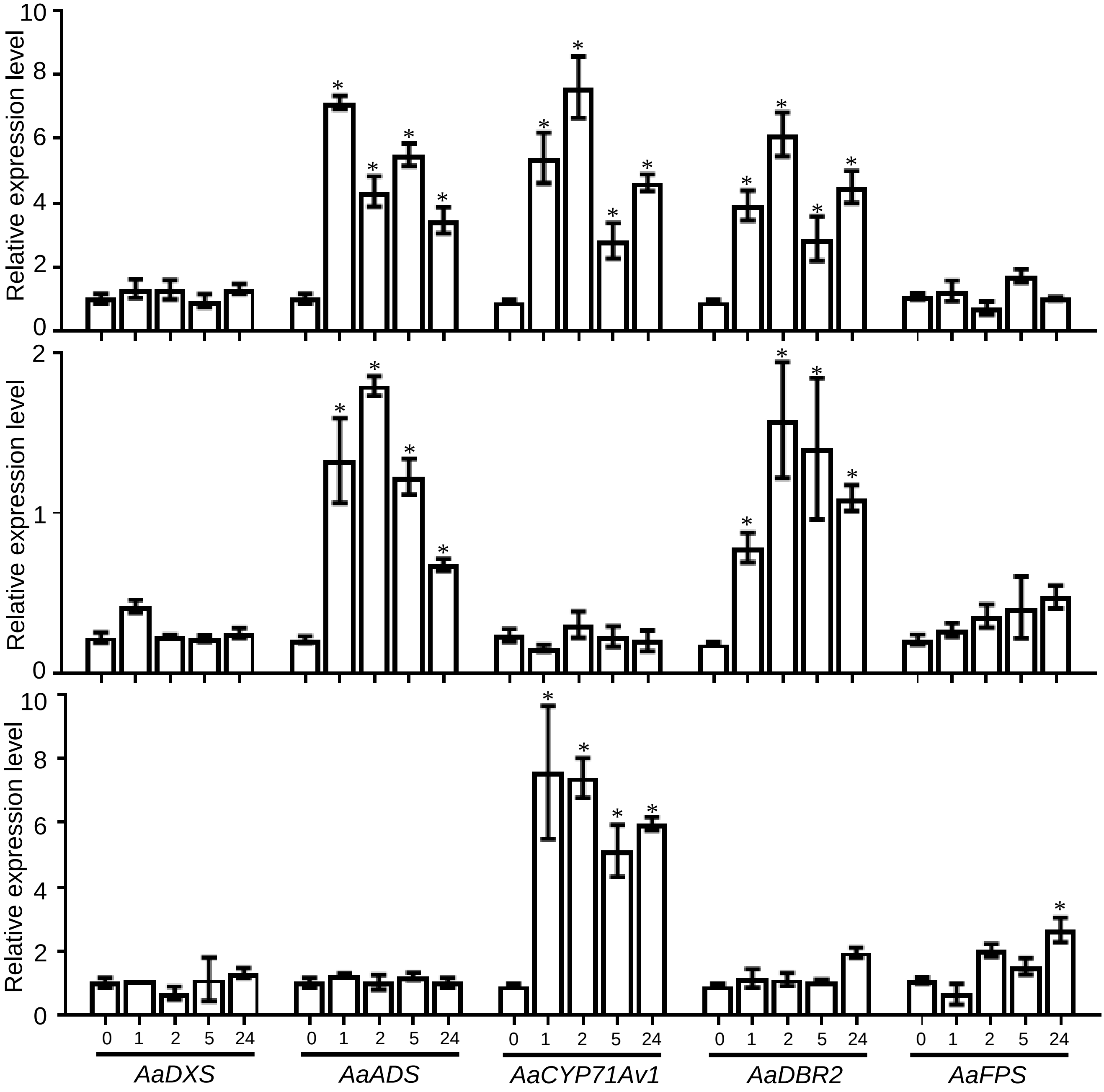

Fig. S4 qRT-PCR analyses of key genes in the artemisinin biosynthetic pathway under the treatments of three different abiotic stresses.

The transcript levels of *AaDXS, AaADS, AaCYP71AV1, AaDBR2* and *AaFPS* genes under cold, NaCl and ABA treatments and mock were normalized. The average ± SD values from three biological repeats are shown. * P < 0.05, Student's *t*-test.
